# Wnt/β-catenin interacts with the FGF pathway to promote proliferation and regenerative cell proliferation in the zebrafish lateral line neuromast

**DOI:** 10.1038/s12276-019-0247-x

**Published:** 2019-05-23

**Authors:** Dongmei Tang, Yingzi He, Wenyan Li, Huawei Li

**Affiliations:** 1 ENT institute and Otorhinolaryngology Department of Affiliated Eye and ENT Hospital, State Key Laboratory of Medical Neurobiology, Fudan University, Shanghai, 200031 China; 2 Institutes of Biomedical Sciences, Fudan University, Shanghai, 200032 China; 3 NHC Key Laboratory of Hearing Medicine, Fudan University, Shanghai, 200031 China; 4 Shanghai Engineering Research Centre of Cochlear Implant, Shanghai, 200031 China; 5 The Institutes of Brain Science and the Collaborative Innovation Center for Brain Science, Fudan University, Shanghai, 200032 China

**Keywords:** Hair cell, Hair cell, Growth factor signalling, Growth factor signalling

## Abstract

Wnt and FGF are highly conserved signaling pathways found in various organs and have been identified as important regulators of auditory organ development. In this study, we used the zebrafish lateral line system to study the cooperative roles of the Wnt and FGF pathways in regulating progenitor cell proliferation and regenerative cell proliferation. We found that activation of Wnt signaling induced cell proliferation and increased the number of hair cells in both developing and regenerating neuromasts. We further demonstrated that FGF signaling was critically involved in Wnt-regulated proliferation, and inhibition of FGF abolished the Wnt stimulation-mediated effects on cell proliferation, while activating FGF signaling with basic fibroblast growth factor (bFGF) led to a partial rescue of the proliferative failure and hair cell defects in the absence of Wnt activity. Whole-mount in situ hybridization analysis showed that the expression of several FGF pathway genes, including *pea3* and *fgfr1*, was increased in neuromasts after treatment with the Wnt pathway inducer BIO. Interestingly, when SU5402 was used to inhibit FGF signaling, neuromast cells expressed much lower levels of the FGF receptor gene, *fgfr1*, but produced increased levels of Wnt target genes, including *ctnnb1*, *ctnnb2*, and *tcf7l2*, while bFGF treatment produced no alterations in the expression of those genes, suggesting that *fgfr1* might restrict Wnt signaling in neuromasts during proliferation. In summary, our analysis demonstrates that both the Wnt and FGF pathways are tightly integrated to modulate the proliferation of progenitor cells during early neuromast development and regenerative cell proliferation after neomycin-induced injury in the zebrafish neuromast.

## Sensory cells: Hearing lessons from signals in fish

Studying sensory organs on the skin of zebrafish is revealing details of molecular signaling pathways that may be relevant to our own sensory systems, especially the hair cells of the ear. These cells have fine hair-like structures that move in response to sound waves and help generate electrical signals to the brain that result in perception of sound. Huawei Li and colleagues at Fudan University, Shanghai, China, studied the roles of two well-known cellular signaling pathways in regulating the proliferation of similar sensory hair cells in zebrafish, a commonly used model organism. These pathways involve cell surface proteins that interact with small extracellular molecules to stimulate molecular changes within cells. Learning how the pathways control hair cell generation and multiplication may assist modification of similar systems in humans to study and treat hearing loss.

## Introduction

Cell proliferation is a critical process guiding many aspects of auditory organ specification and morphogenesis during embryonic development, and its misregulation is associated with various malformations. However, the precise molecular signaling pathways that control proliferation and their coordination remain unclear.

The zebrafish lateral line is a powerful vertebrate model system for the in vivo study of sensory organ developmental biology due to its well-established genetics and ease of in vivo visualization and manipulation. The posterior lateral line (pLL) comprises a series of mechanosensory organs called neuromasts, which lie on the surface of both the head and body. Neuromasts are composed of sensory hair cells (HCs) in the center that are surrounded by nonsensory supporting cells (SCs)^1,2^. Mechanosensory HCs share many structural, molecular, and functional similarities with vertebrate inner ear HCs and thus are an excellent model system for studying HC biology related to hearing and balance and for unraveling their genetic control^3,4^. However, unlike mammals, zebrafish retain the ability to quickly regenerate HCs after damage^5–8^. Thus, the molecular signaling pathways that guide HC regeneration in the zebrafish lateral line are of great biological and clinical interest.

Previous studies have revealed that the development of the pLL requires complex coordination among diverse molecular signaling pathways, including chemokine signaling^9–11^, FGF signaling^11–13^, and Wnt/β-catenin signaling^10,11^. Most of our current understanding about these pathways is related to early embryonic pLL formation, which entails primordium formation, migration, and deposition stages; however, the precise molecular signaling that initiates and guides cell proliferation and HC regeneration in zebrafish neuromasts remains poorly defined. The Wnt/β-catenin signaling pathway is a well-known signaling cascade that plays central roles in embryonic development and organogenesis by modulating cell migration, proliferation, and specification^14^. During the early stages of zebrafish pLL development, Wnt/β-catenin signaling is active in the leading part of the primordium and is necessary for cell proliferation and proneuromast formation as the primordium migrates along the body of the organism^15–17^. Constitutive activation of canonical Wnt signaling broadly promotes cellular proliferation throughout the primordium but stalls migration during developmental patterning, whereas pharmacological or genetic inhibition of Wnt reduces cellular proliferation, increases cell death, and results in a dramatic truncation of the pLL^10,11,18^. Recently, activation of the Wnt/β-catenin pathway was reported to promote proliferation and increase HC generation in the developing and regenerating zebrafish lateral line^19^. Conversely, inhibition of canonical Wnt signaling decreases proliferation and arrests neuromast morphogenesis at an early stage of neuromast development. Little is known, however, about the molecular mechanisms underlying this signal transduction during the periods of proliferation and HC regeneration in neuromasts.

FGF signaling has been shown to regulate cell migration and cell fate changes during embryonic development^20^, and FGF is active in the trailing region of the zebrafish pLL primordium, which contains two or three rosette-shaped proneuromasts. FGF acts as a key molecule in promoting the epithelialization and formation of the proneuromasts in the course of the migration of the primordium^12,13^, and FGF inhibition leads to a loss of apically constricted rosettes and aberrant migration of the primordium. FGF activation not only induces pro-neuromast formation by initiating the organization of center-oriented epithelial rosettes but also initiates the specification of HC precursors by regulating *atoh1a* expression^12,13^. Although the function of FGF signaling in facilitating pLL morphogenesis has been reasonably well studied, its contribution to proliferation during the establishment of the pLL and to regenerative cell proliferation has not been well addressed.

In this study, we used pharmacological agonists and antagonists and transgenic zebrafish to regulate Wnt and FGF signaling, and we found significantly more proliferating cells and more HCs and SCs after activation of Wnt or FGF signaling. To further understand whether the Wnt and FGF pathways act synergistically to regulate proliferation, we performed epistasis experiments to verify the mechanisms underlying proliferation in neuromasts. We stimulated Wnt signaling first and then inhibited FGF activity and found that the proliferation of the progenitors induced by Wnt activation disappeared after blocking FGF in Wnt-activated embryos. Conversely, we found that inhibition of Wnt signaling using the DKK1 conditional knockout transgenic line or IWR-1 treatment followed by treatment with basic fibroblast growth factor (bFGF) resulted in significantly more proliferating cells than in the group treated with IWR-1 alone, suggesting that activation of FGF could partly rescue proliferation failure caused by Wnt signaling inhibition. Similar results were observed in HC regeneration experiments. To better understand the interactions of both signaling pathways during cell proliferation in the larval zebrafish neuromast, we performed whole-mount in situ hybridization analysis. We showed that the *fgf3* and *fgf10* genes were Wnt targets during neuromast cell proliferation and acted to modulate FGF activity and that *fgfr1*, an FGF target gene, acted to repress Wnt activity. Taken together, our findings suggest that the Wnt and FGF signaling pathways are tightly connected to regulate developmental cell proliferation and regenerative cell proliferation in the zebrafish pLL neuromast.

## Materials and methods

### Fish strains and maintenance

Embryos were obtained by natural spawning and developed at 28.5 °C in E3 medium. They were staged according to standard protocols^21^, and embryo ages were marked as hours post fertilization (hpf). The wild-type strain was derived from the AB line, and the *Tg(brn3c:mGFP)*^*s356t*^ line was used to visualize HCs. The *apc*^*mcr*^ line was a generous gift from Professor Xu Wang. APC genotyping primers were as follows: APCR—CAT GGC TCA CTC TGC ACA; APCWTF—ATA ATG TTG CAG CTG ACC; and APCMTF—ATA ATG TTG CAG CTG ACT. *Tg(hsp70l:dkk1b-GFP)* offspring were incubated at 42 °C for 5 min at 48 hpf and allowed to recover at 28.5 °C to inhibit Wnt signaling. Approximately 50% of the embryos did not turn green, and these served as controls. To prevent pigment formation, embryos were treated with 0.003% 1-phenyl-2-thiourea (PTU, Sigma-Aldrich, St. Louis, MO, USA) in E3 water from 14 hpf onwards. The larvae were anesthetized in 0.02% MS-222 (Sigma-Aldrich, Inc.) before fixation. All zebrafish experiments were performed following the institutional guidelines approved by the Institutional Animal Care and Use Committee of Fudan University, Shanghai.

### Pharmacological treatment

We used 1 μM BIO to activate Wnt signaling and 10 μM IWR-1 (Sigma-Aldrich) to inhibit Wnt signaling, and we used 5 μM SU5402 (Calbiochem) to inhibit FGF signaling and 20 ng/ml bFGF (Invitrogen) to activate FGF signaling. Neomycin sulfate (Sigma-Aldrich) was added to a final concentration of 400 μM, and the 5 dpf larvae were incubated for one hour, followed by three rinses in fresh egg water. The larvae were then allowed to recover at 28.5 °C.

### BrdU incorporation and immunohistochemistry

For immunofluorescence experiments, 10 mM BrdU (Sigma-Aldrich) was coincubated with the pharmacological treatments described above to label the proliferating cells. Zebrafish larvae were fixed with 4% PFA for 2 h at room temperature and washed three times with PBT-2 (PBS containing 1% Triton X-100). For DNA denaturing, the fixed larvae were treated with 2 N HCl for 30 min at 37 °C followed by three rinses with PBT-2. Before antibody staining, the larvae were incubated in blocking solution (10% donkey serum in PBT-2) for 1 h at 37 °C. The primary antibodies were anti-Sox2 (1:200 dilution; Abcam), anti-BrdU (1:200 dilution; Santa Cruz Biotechnology), anti-GFP (1:500 dilution; Abcam), and anti-Myosin VI (1:200 dilution; Proteus BioSciences). After incubation with primary antibodies overnight at 4 °C, the larvae were washed three times with PBT-2 and then incubated with Alexa Fluor 488-, 594-, and/or 647-conjugated secondary antibodies (1:200 dilution; Jackson ImmunoResearch Laboratories, West Grove, PA, USA) for 1 h at 37 °C. After the larvae were washed several times in PBT-2, they were incubated with DAPI (1:800 dilution; Invitrogen) for 20 min to label the nuclei.

### Imaging and cell counts

Fluorescent specimens were examined using a Leica confocal microscope (TCS SP8; Leica, Wetzlar, Germany). All images were edited using Adobe Photoshop CS6. The labeled cells in the pLL neuromasts (pLL2–pLL5) were counted in the confocal and fluorescence images using the counting and measuring tools in Adobe Photoshop CS6.

### Whole-mount in situ hybridization

Digoxigenin-labeled probes were prepared as recommended by the manufacturer (Roche, Mannheim, Germany). Primers for cloning the examined genes are listed in Table S1. Regular whole-mount in situ hybridization (WISH) of zebrafish embryos was performed as previously described^22^. After the color reaction, the embryos were mounted with 100% glycerol and observed under a bright field microscope (Nikon Instruments). Binding sites were identified as blue-black dots.

### TUNEL staining

Apoptotic cells in the trunk of the zebrafish were detected by TUNEL assay (In Situ Cell Death Detection Kit, Roche). After the larvae were rinsed three times with PBT-2, they were incubated with the TUNEL reaction mixture in a humid dark chamber at 37 °C for 1 h and then labeled with DAPI to visualize the nuclei.

### Statistical analysis

All statistical analyses were performed with GraphPad Prism 6 Software (GraphPad, San Diego, CA, USA). Cell counts were analyzed using Student’s *t*-tests and one-way ANOVA. In all figures, error bars represent the mean ± SEM. *p* < 0.05 was considered statistically significant, and *p* < 0.001 was considered highly significant. *Shows the comparison with controls, and ^**#**^ is used when the Wnt and FGF signaling pathways were shown to interact.

## Results

### The role of Wnt/β-catenin signaling in the developing neuromast of the zebrafish pLL

To determine in detail how Wnt/β-catenin signaling affects early neuromast development, BIO and IWR-1 were used to modulate Wnt activity at 48 hpf when the neuromasts along the trunk and tail had all been deposited and the neuromast cells were undergoing high levels of proliferation. The effects of the treatments were detected by WISH with known markers of Wnt signaling, including *ctnnb1*, *ctnnb2*, and *tcf7l2*. After 24 h of pharmacological treatment, a significant increase in *ctnnb1*, *ctnnb2*, and *tcf7l2* expression was observed in the 1 μM BIO-treated group, indicating induction of Wnt signaling, whereas 10 μM IWR-1 significantly inhibited the expression of these genes (Supplementary Fig. 1). Thus, 1 μM BIO and 10 μM IWR-1 were used for all subsequent experiments in this study.

**Fig. 1:**
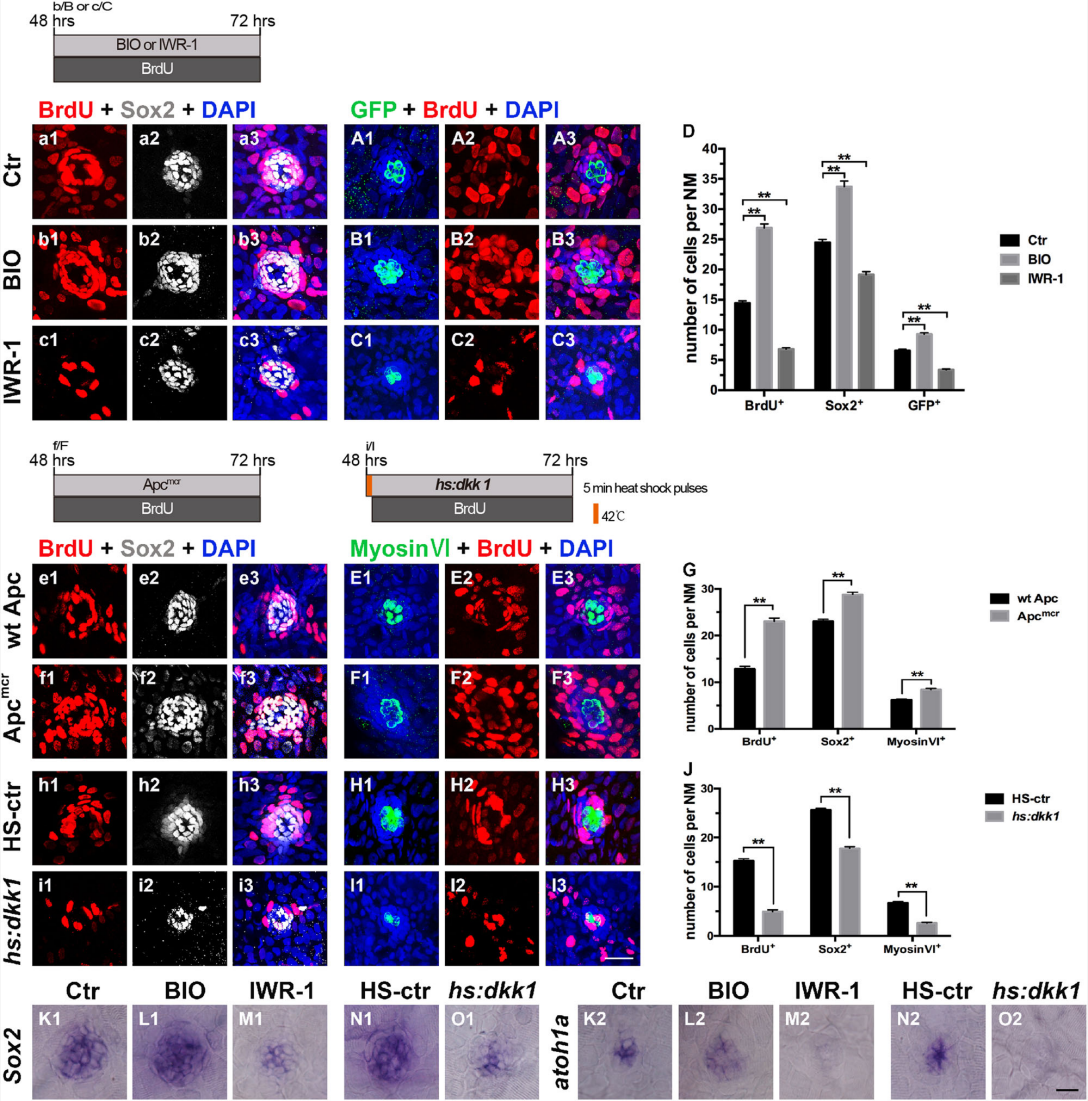
Effects of Wnt signaling on cell proliferation in the developing neuromast. **a1**–**C3**: Proliferative cells labeled with BrdU (red), SCs labeled with Sox2 (white), and HCs labeled with GFP (green). **a1**–**A3**: Control neuromasts; **b1**–**B**3: After addition of BIO; **c1**–**C3**: After addition of IWR-1. **d**: Quantification of proliferative cells (BrdU^+^), SCs (Sox2^+^), and HCs (GFP^+^) shown in **a1**–**C3**. **e1**–**F3** and **h1**–**I3**: Proliferative cells labeled with BrdU (red), SCs labeled with Sox2 (white), and HCs labeled with myosin VI (green). **e1**–**E3**: wt Apc larvae; **f1**–**F3**: Apc^mcr^ mutants showing elevated Wnt/β-catenin activity; **h1**–**H3**: Heat shock-negative control larvae (HS-ctr); **i1**–**I3**: Wnt repression by heat shock induction of the *hs:dkk1* transgene (*hs:dkk1*). **g**, **j**: Quantification of the proliferative cells (BrdU^+^), SCs (Sox2^+^), and HCs (Myosin VI^+^) shown in **e1**–**F**3 (**g**) and **h1**–**I3** (**j**). *n* = 5–7 fish per group. ** Indicates *p* < 0.001, and error bars indicate the standard error of the mean. **K1**–**O1** and **K2–O2:** WISH analysis of *Sox*2 (**K1–O1**) and *atoh1a* (**K2–O2**) expression in the neuromasts from different groups. Scale bar in **I3** **=** 20 μm for **a1–C3**, **e1–F3,** and **h1–I3.** Scale bar in **O2** = 30 μm for **K1**–**O1**, **K2**–**O2**

To simplify the identification of neuromast cells, we used the *tg*(*Brn3c:mGFP*) transgenic line that expresses GFP in the HC membrane^23^, and the SCs were marked and counted by Sox2 immunostaining. Beginning at 48 hpf, embryos were treated with BIO or IWR-1 in the presence of BrdU for a period of 24 h. Quantification showed significantly more BrdU^+^ cells, GFP^+^ HCs, and Sox2^+^ SCs in the BIO-treated neuromasts than in the DMSO-treated control neuromasts (Fig. 1a1–a3, b1–b3, A1–A3, B1–B3). In contrast, IWR-1-treated embryos showed significantly fewer BrdU^+^ cells, HCs, and SCs in the developing neuromasts than did DMSO-treated controls (Fig. 1c1–c3, C1–C3, d), which is consistent with previous results^19^. Additionally, no significant change in apoptosis was observed after activation or suppression of Wnt activity (Supplementary Fig. 3).

To further verify the role of Wnt/β-catenin signaling in neuromast development, we analyzed mutants and transgenic fish that respectively increase or decrease Wnt/β-catenin signaling. The first was a recessive zebrafish mutation in the *apc* gene (referred to here as Apc^mcr^)^24,25^, and the second was a heat-shock–inducible *dkk1* transgenic line (*hsp70l:dkk1b-GFP*, referred to here as *hs:dkk1*)^26^. In these experiments, myosin VI was used to label mature HCs. Consistent with the pharmacological results, the numbers of proliferating cells and differentiated cells (HCs and SCs) in neuromasts were significantly higher in Apc^mcr^ larvae relative to those in wild-type (wt Apc) at 72 hpf (Fig. 1e1–e3, E1–E3, f1–f3, F1–F3, g), whereas heat-shocked *dkk1b-GFP* larvae had significantly fewer proliferating cells and differentiated cells than nonheat-shocked controls (HS-ctr) (Fig. 1h1–h3, H1–H3, i1–i3, I1–I3, j). WISH results showed that activating Wnt produced a significant increase in the mRNA level of the SC marker *sox2* and the HC marker *atoh1a*, whereas inhibition of Wnt signaling caused downregulation of both of genes compared with that observed in controls (Fig. 1K1–O1 and K2–O2).

### FGF signaling promotes cell proliferation and is required for HC differentiation

FGF signaling is well known to be involved in multiple developmental processes, including normal otic placode formation and maintenance as well as pLL formation^10,12,20,27,28^. We, therefore, examined whether FGF signaling plays an important role in neuromast development in the zebrafish pLL. We first conducted a comprehensive expression analysis by means of WISH to examine the expression of known FGF signaling-related genes. In 72 hpf zebrafish larvae, expression of the FGF signaling receptor gene (*fgfr1*) and target gene (*pea3*) was clearly detectable within the neuromast (Supplementary Fig. 2A1–A2), indicating that this pathway is involved in early developmental processes in the neuromast. We studied the role of FGF signaling in neuromast development by blocking FGF with the small molecule SU5402, a specific FGF receptor tyrosine kinase inhibitor^29^. We treated 48 hpf embryos with SU5402 at varying concentrations (5, 10, or 15 μM) for 24 h, with DMSO treatment as the control. At the highest dose (15 μM), we observed evident larval death (10/12), whereas 5 μM SU5402 did not cause developmental abnormalities or death. We then measured the expression of *pea3* and *fgfr1*, and WISH showed that the expression of both genes was significantly downregulated after 5 μM SU5402 treatment for 24 h compared to expression in DMSO controls (Supplementary Fig 2A1–A2 and B1–B2). Thus, we used 5 μM SU5402 in subsequent experiments.

**Fig. 2:**
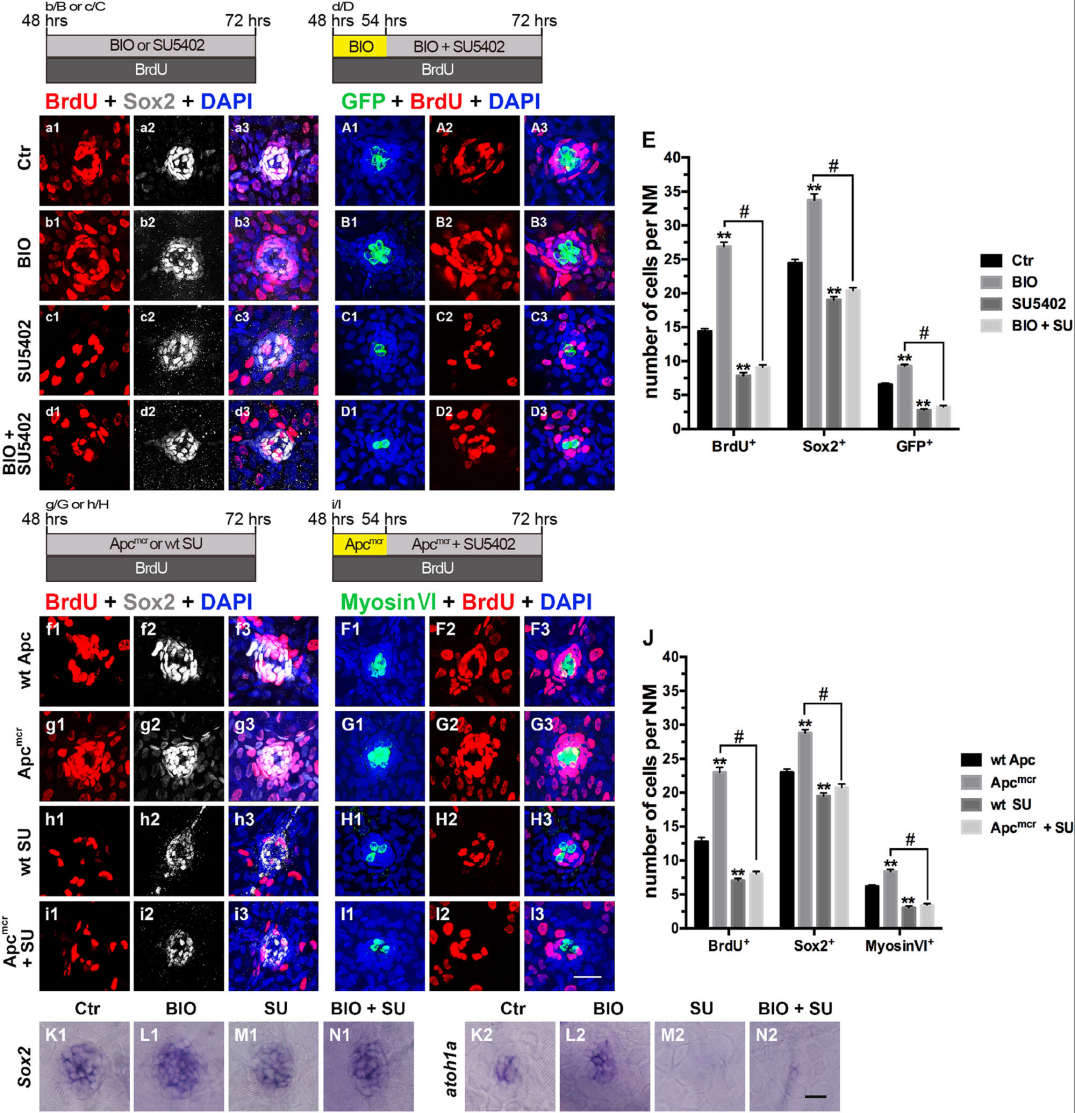
Effects of the Wnt and FGF signaling pathways on proliferation and differentiation during initial neuromast development. **a1**–**D3**: Proliferative cells labeled with BrdU (red), SCs labeled with Sox2 (white), and HCs labeled with GFP (green). **a1**–**A3**: Control neuromast; **b1**–**B**3: After addition of BIO; **c1**–**C3**: After addition of SU5402; **d1**–**D3**: After 6 h of incubation with BIO, SU5402 and BIO were added for 18 h; **e**: Quantification of the proliferative cells (BrdU^+^), SCs (Sox2^+^), and HCs (GFP^+^) shown in **a1**–**D3**. **f1**–**I3**: Proliferative cells labeled with BrdU (red), SCs labeled with Sox2 (white), and HCs labeled with myosin VI (green). **f1**–**F3**: wt Apc larvae; **g1**–**G3**: Apc^mcr^ mutants; **h1**–**H3**: After addition of SU5402; **i1**–**I3**: 54 hpf Apc^mcr^ embryos were treated with SU5402 for 18 h. **j**: Quantification of the proliferative cells (BrdU^+^), SCs (Sox2^+^), and HCs (Myosin VI^+^) shown in **f1**–**I3**. *n* = 5–7 fish per group. ** Indicates *p* < 0.001, ^#^ indicates *p* < 0.05, and error bars indicate the standard error of the mean. **K1**–**N1** and **K2**–**N2**: WISH analysis of *Sox*2 (**K1**–**N1**) and *atoh1a* (**K2**–**N2**) expression in the neuromasts from different groups. Scale bar in I3 = 20 μm for **a1**–**D3** and **f1**–**I3**. Scale bar in **N2** = 30 μm for **K1**–**N1**, **K2**–**N2**

Quantification and comparison of BrdU^+^ cells in the SU5402-treated larvae and DMSO-treated controls showed a significant reduction in the number of proliferating cells generated after Fgfr blockade (Fig. 2a1, a3, c1, c3, A2–A3, C2–C3, e), which was similar to inhibition of Wnt signaling. Loss of FGF signaling also resulted in a significant decrease in the development of neuromasts, as indicated by the robust loss of Sox2^+^ SCs and GFP^+^ HCs (Fig. 2a2–a3, A1, A3, c2–c3, C1, C3, e).

### Wnt/β-catenin signaling acts upstream of FGF signaling in neuromast development

Because both Wnt/β-catenin and FGF signaling components are expressed in the developing neuromast and are required for cell proliferation and HC differentiation, we analyzed the potential epistasis between Wnt and FGF signaling. First, we treated 48 hpf embryos with 1 μM BIO alone for 6 h to activate Wnt/β-catenin signaling. Then, we added 5 μM SU5402 for 18 h along with BIO to inhibit FGF activity, which was compared to BIO treatment alone for 24 h. Interestingly, the BIO-induced cell proliferation along with the increasing number of SCs and HCs was significantly disrupted by the FGFR1 antagonist SU5402 (Fig. 2b1– b3, B1–B3, d1–d3, D1–D3, e). We found no significant differences between the BIO+SU5402 group and the SU5402 alone for 24 h group (Fig. 2c1–c3, C1–C3, d1–d3, D1–D3, e).

The same results were obtained when we used transgenic Apc^mcr^ embryos instead of BIO to activate Wnt/β-catenin signaling. At 54 hpf, Apc^mcr^ embryos were incubated in 5 μM SU5402 for 18 h, and the wt Apc and wt SU5402 groups served as controls (Fig. 2f1–f3, F1–F3, h1–h3, H1–H3). When treated with SU5402, the increased numbers of BrdU^+^, Sox2^+^, and Myosin VI^+^ cells in the neuromasts of Apc^mcr^ larvae were all reduced (Fig. 2g1–g3, G1–G3, i1–i3, I1–I3, j). Similarly, there were no significant differences in the numbers of BrdU^+^, Sox2^+^, or Myosin VI^+^ cells between the Apc^mcr^ +SU5402 group and wt SU5402 group (Fig. 2h1–h3, H1–H3, i1–i3, I1–I3, j). Inhibiting FGF signaling caused downregulation of *sox2* and *atoh1a* expression (Fig. 2M1 and M2) compared with control treatment (Fig. 2K1 and K2), whereas activation of Wnt followed by inhibition of FGF signaling led to a significant reduction in the mRNA level of both genes (Fig. 2N1 and N2) when compared with the level in the Wnt activation alone group (BIO) (Fig. 2L1 and L2).

**Fig. 3:**
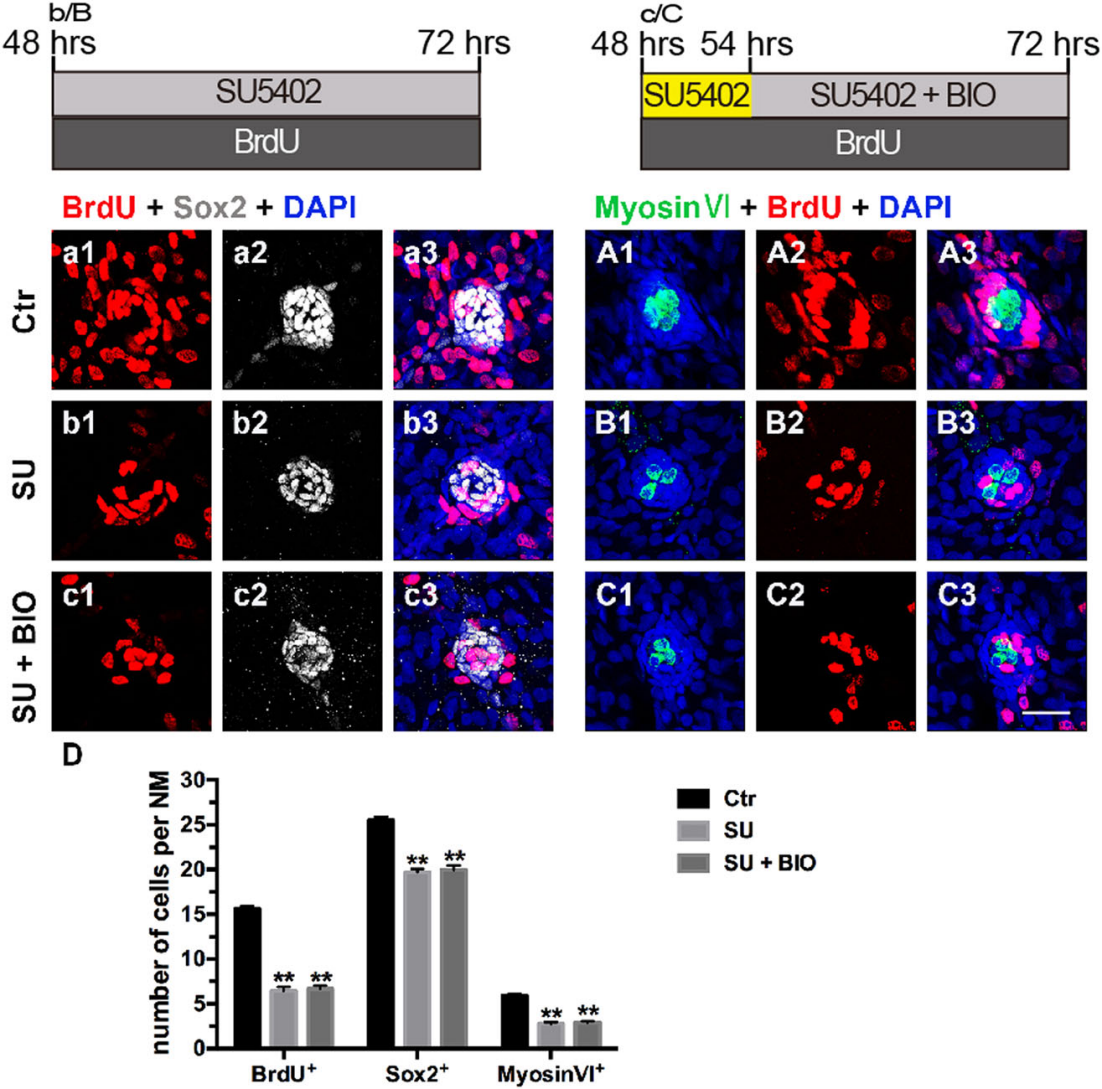
Effects of Wnt activation on cell proliferation and differentiation in FGF-inhibited larvae. **a1**–**C3**: Proliferative cells labeled with BrdU (red), SCs labeled with Sox2 (white), and HCs labeled with myosin VI (green). **a1**–**A3**: Control neuromast; **b1**–**B3**: After addition of SU5402; **c**1–**C3**: After 6 h of incubation with SU5402, BIO and SU5402 were added for 18 h; **d**: Quantification of the proliferative cells (BrdU^+^), SCs (Sox2^+^), and HCs (Myosin VI^+^) shown in **a1**–**C3**. *n* = 5–7 fish per group. ** Indicates *p* < 0.001, and error bars indicate the standard error of the mean. Scale bar in C3 = 20 μm for **a1**–**C3**

Furthermore, we treated 48 hpf wild-type embryos with SU5402 for 24 h to inhibit FGF signaling or treated them with SU5402 for 6 h first and then coincubated with BIO for another 18 h. DMSO-treated larvae were used as controls. We found that the numbers of proliferating cells and differentiated cells were significantly reduced in SU5402-treated larvae compared with those in the controls and that BIO treatment had no effect on the reduced numbers of proliferating and differentiated cells in the neuromasts caused by SU5402 (Fig. 3).

### The reduced proliferation induced by Wnt/β-catenin inhibition is partially rescued by upregulation of FGF signaling

**Fig. 4:**
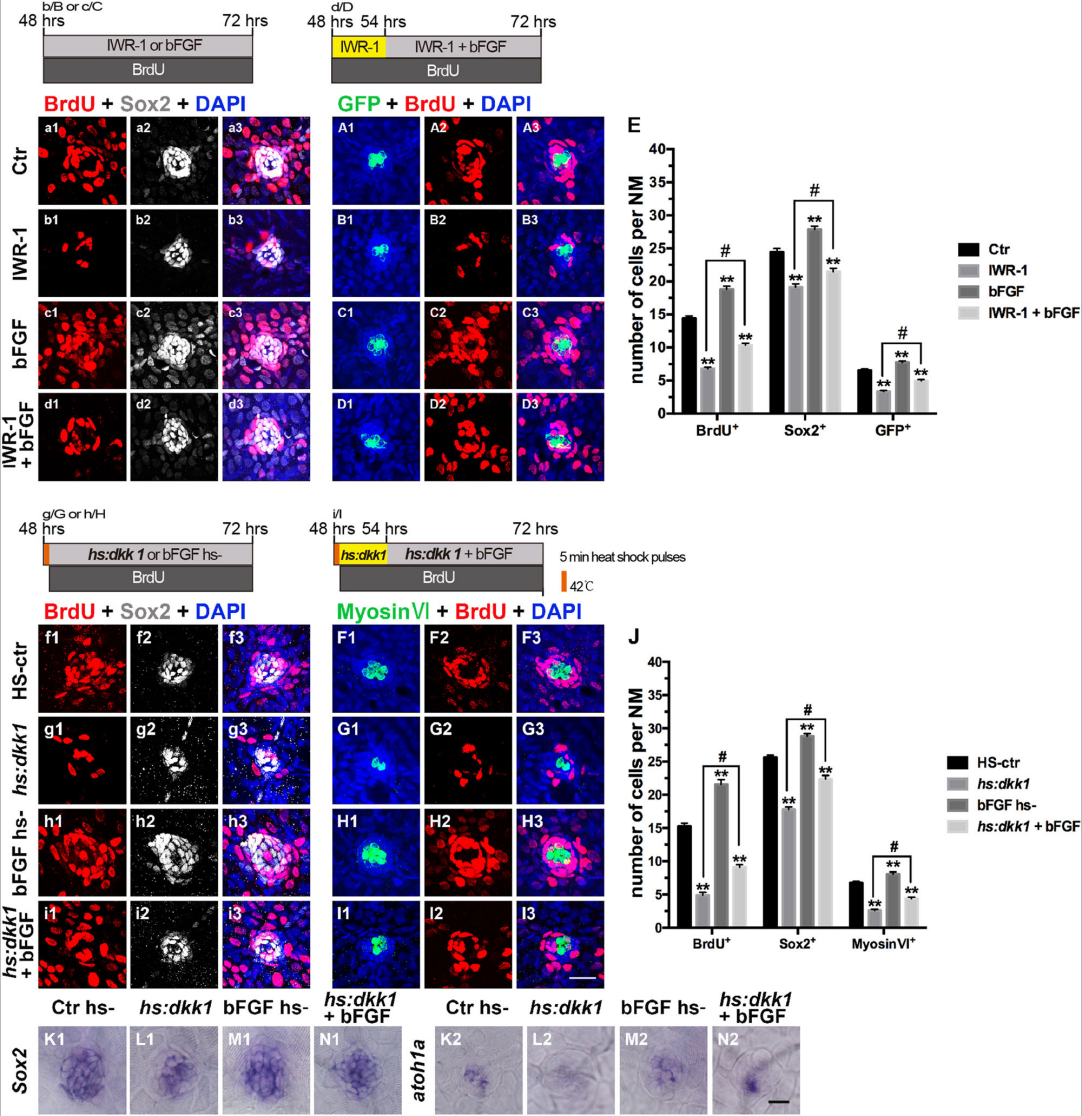
Effects of FGF activation on cell proliferation in Wnt-inhibited larvae. **a1**–**D3**: Proliferative cells labeled with BrdU (red), SCs labeled with Sox2 (white), and HCs labeled with GFP (green). **a**1–**A**3: Control neuromast; **b1**–**B3**: After addition of IWR–1; **c1**–**C3**: After addition of bFGF; **d1**–**D3**: After 6 h of incubation with IWR–1, bFGF and IWR–1 were added for 18 h; **e**: Quantification of the proliferative cells (BrdU^+^), SCs (Sox2^+^), and HCs (GFP^+^) shown in **a1**–**D3**. **f1**–**I3**: Proliferative cells labeled with BrdU (red), SCs labeled with Sox2 (white), and HCs labeled with myosin VI (green). **f1**–**F3**: Heat shock-negative control larvae (HS–ctr); **g1**–**G3**: Wnt repression by heat shock induction of the *hs:dkk1* transgene (*hs:dkk1*); h1–H3: After addition of bFGF; **i1**–**I3**: At 54 hpf, *hs:dkk1* embryos were treated with bFGF for 18 h. **j**: Quantification of the proliferative cells (BrdU^+^), SCs (Sox2^+^), and HCs (myosin VI^+^) shown in **f1**–**I3**. *n* = 5–7 fish per group. ** Indicates *p* < 0.001, ^#^ indicates *p* < 0.05, and error bars indicate the standard error of the mean. **K1**–**N1** and **K2**–**N2**: WISH analysis of *Sox*2 (**K1**–**N1**) and *atoh1a* (**K2**–**N2**) expression in the neuromasts from different groups. Scale bar in **I3** = 20 μm for **a1**–**D3** and **f1**–**I3**. Scale bar in **N2** = 30 μm for **K1**–**N1**, **K2**–**N2**

Given the expression of *fgfr1* in the neuromast and the critical role of FGF signaling during neuromast development in zebrafish, FGF activation might be responsible for cell proliferation. To test this possibility, we treated 48 hpf zebrafish larvae with bFGF, also known as FGF-2, which is able to bind Fgfr1-3 and is involved in various biological processes, such as cell proliferation, angiogenesis, differentiation, and tumor development^30–32^. After treatment with bFGF (20 ng/ml) for 24 h, the expression of *pea3* and *fgfr1* was robustly upregulated compared to the expression in unstimulated neuromasts, and no deformities were detected (Supplementary Figs 2A1–A2 and C1–C2). Furthermore, the addition of bFGF (20 ng/ml) increased cell proliferation and differentiation (Fig. 4a1–a3, A1–A3, c1–c3, C1–C3, e) and enhanced expression of *atoh1a* in neuromasts (Fig. 4K2 and M2).

To further confirm the relationship between Wnt and FGF signaling, we treated 48 hpf embryos with IWR-1 for 6 h and then incubated them in the presence or absence of bFGF for another 18 h. Interestingly, significantly more BrdU^+^ cells were observed in the neuromasts of the IWR-1+bFGF group than in those of the IWR-1 only group, indicating that bFGF treatment partly rescued the proliferative defect caused by IWR-1 inhibition (Fig. 4b1, b3, B2–B3, d1, d3, D2–D3, e). Furthermore, the IWR-1 + bFGF-treated group had significantly more Sox2^+^ SCs and GFP^+^ HCs than the IWR-1–alone group (Fig. 4b2–b3, B1, B3, d2–d3, D1, D3, e) but fewer than the controls (Fig. 4a2–a3, A1, A3, e). Similar results were obtained when we heat shocked *dkk1b-GFP* transgenic embryos at 48 hpf and then incubated them in 20 ng/ml bFGF (Fig. 4f1–f3, F1–F3, g1–g3, G1–G3, h1–h3, H1–H3, i1–i3, I1–I3, j). These experiments indicated that activating FGF signaling could partly rescue the proliferation failure caused by inhibiting Wnt/β-catenin signaling, suggesting that FGF signaling acts downstream of Wnt/β-catenin signaling. Although inhibiting Wnt signaling decreased the expression of *sox2* and *atoh1a* (Fig. 4K1, L1, K2, and L2), bFGF treatment rescued the decreased expression of *sox2* and *atoh1a* induced by blocking Wnt signaling (Fig. 4L1–N1 and L2–N2).

### Wnt and FGF signaling are integrated to regulate proliferation during development

**Fig. 5:**
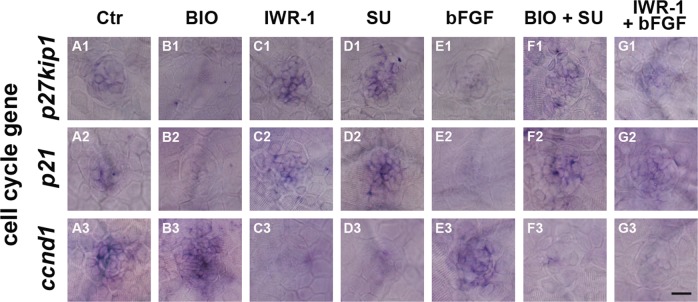
Whole-mount in situ hybridization of *p27*, *p21*, and *ccnd1* following regulation of Wnt and FGF signaling. **A1**–**G3** In situ hybridization of *p27* (**A1**–**G1**) *p21* (**A2**–**G2**) and *ccnd1* (**A3**–**G3**) following BIO (**B1**–**B3**), IWR-1 (**C1**–**C3**), SU5402 (**D1**–**D3**), bFGF (**E1**–**E3**), BIO+SU5402 (**F1**–**F3**), and IWR-1+bFGF (**G1**–**G3**) treatments. Scale bar in **G3** = 30 μm for **A1**–**G3**

Because one important role of both the Wnt and FGF signaling pathways during neuromast development is to promote cell proliferation, we focused on the cell cycle genes *p27*, *p21*, and *ccnd1* as possible downstream mediators of Wnt and FGF activity. Activation of FGF signaling didn’t change the expression of *ccnd1* significantly but downregulated the expression of the cell cycle inhibitors *p27* and *p21* as shown in Fig. 5E1–E3, whereas blockage of FGF signaling using the FGFR inhibitor SU5402 resulted in a near complete loss of *ccnd1* expression and an increase in *p27* and *p21* expression compared to control treatment (Fig. 5D1–D3 and A1–A3, respectively). When Wnt signaling was activated by BIO, the expression of *p27* and *p21* was significantly downregulated in the neuromast (Fig. 5B1–B2); however, in the absence of FGF (BIO+SU5402), the expression of both genes was increased, especially the expression of *p21* (Fig. 5F1–F2). Meanwhile, overexpression of Wnt induced the expression of *ccnd1* (Fig. 5B3), and inhibition of FGF led to decreased *ccnd1* levels (Fig. 5F3). In addition, inhibition of Wnt signaling by IWR-1 at 48 hpf resulted in the near complete loss of *ccnd1* expression and an increase in *p27* and *p21* expression at 72 hpf compared to control treatment (Fig. 5A1–A3 and C1–C3, respectively), while inhibition of Wnt followed by activation of FGF signaling led to a slight reduction in the mRNA levels of both the *p27* and *p21* genes when compared with IWR-1 treatment alone (Fig. 5C1–C2 and G1–G2). The activation of FGF signaling didn’t rescue the reduction of *ccnd1* when inhibition of Wnt signaling (Fig. 5C3 and G3). Together, these results suggest that FGF signaling is essential for maintaining the expression of cell cycle genes.

**Fig. 6:**
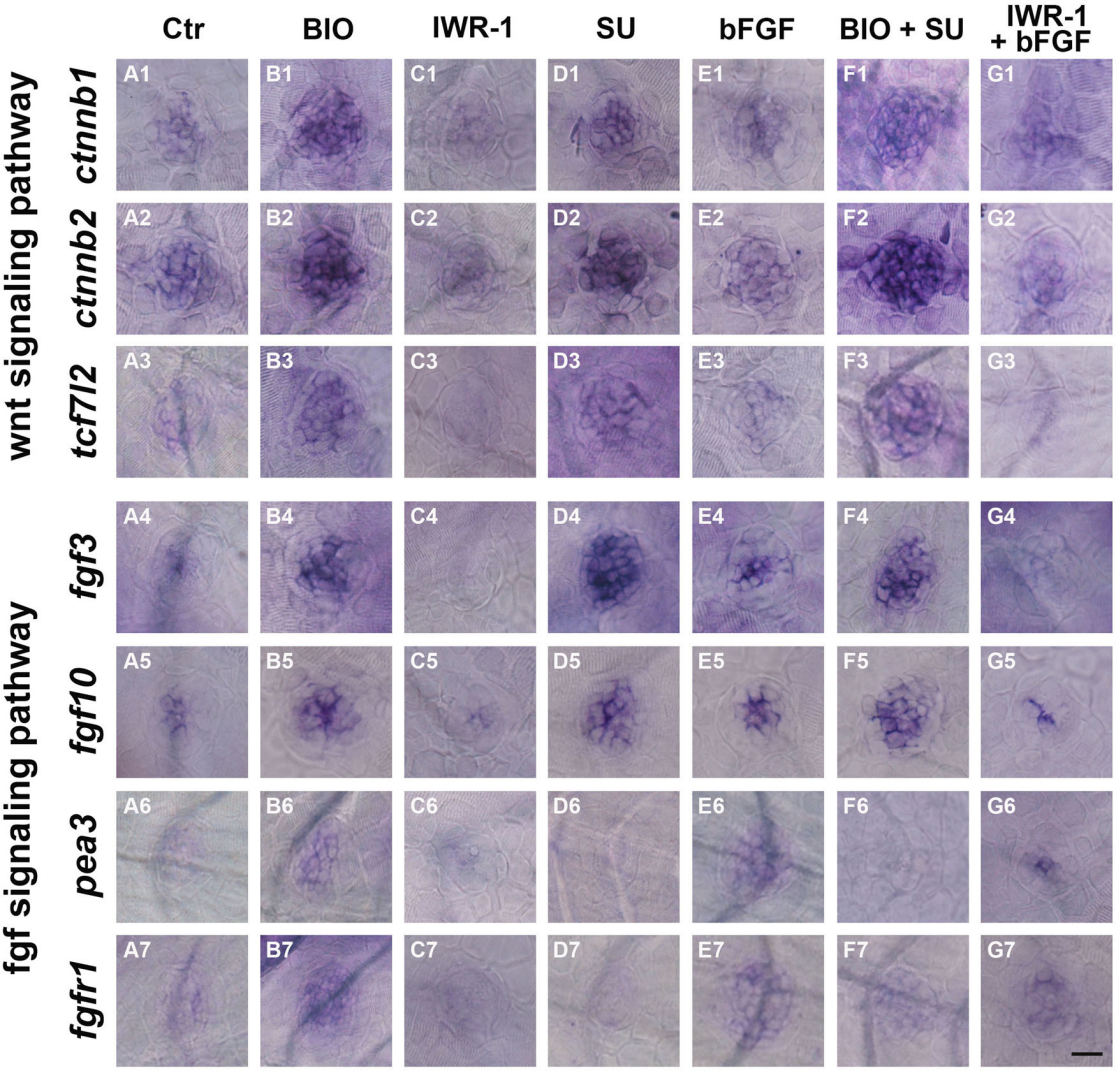
Whole-mount in situ hybridization of several Wnt and FGF target genes following regulation of Wnt and FGF signaling. In situ hybridization of *ctnnb1* (**A1**–**G1**), *ctnnb2* (**A2**–**G2**), *tcf7l2* (**A3**–**G3**), *fgf3* (**A4**–**G4**), *fgf10* (**A5**–**G5**), *pea*3 (**A6**–**G6**), and *fgfr1* (**A7**–**G7**) following BIO (**B1**–**B7**), IWR-1 (**C1**–**C7**), SU5402 (**D1**–**D7**), bFGF (**E1**–**E7**), BIO+SU5402 (**F1**–**F7**), and IWR-1+bFGF (**G1**–G7) treatments. Scale bar in **G7** = 30 μm for **A1**–**G7**

We have shown that FGF signaling is required for cell proliferation in neuromasts and that FGF activity is regulated by Wnt/β-catenin signaling. However, precisely which factors are involved in the interaction between the two pathways is unclear. To define the molecular network between Wnt and FGF during the proliferation of zebrafish neuromasts, we examined neuromasts for the expression of some target genes of the Wnt (*ctnnb1*, *ctnnb2*, and *tcf7l2*) and FGF (*fgf3*, *fgf10*, *pea3*, and *fgfr1*) signaling pathways. As expected, in BIO-treated larvae, the presumed Wnt target genes *ctnnb1*, *ctnnb2*, and *tcf7l2* were markedly upregulated in the neuromast, which was consistent with sustained activation of Wnt/β-catenin signaling, whereas significant decreases were detected for these genes in the IWR-1 treatment group (Fig. 6A1–A3, B1–B3, and C1–C3). Interestingly, Wnt activation significantly increased the expression levels of target genes associated with the FGF signaling pathway, such as *fgf3*, *fgf10*, *pea3*, and *fgfr1* (Fig. 6A4–A7 and B4–B7), whereas the expression of these genes was strongly downregulated in IWR-1-treated larvae (Fig. 6C4–C7). These results further demonstrate that Wnt/β-catenin signaling regulates FGF signaling during proliferation. To further determine how the Wnt and FGF signaling pathways interact, we stimulated FGF signaling with bFGF (Fig. 6E4–E7) and found that Wnt pathway gene expression was unaffected by bFGF treatment (Fig. 6E1–E3) compared to the expression in unstimulated neuromasts (Fig. 6A1–A3), suggesting that FGF signaling is regulated by Wnt signaling during the neuromast developmental stage. However, when SU5402 was used to inhibit FGF signaling, neuromast cells expressed much lower levels of *pea3* and *fgfr1* (Fig. 6D6-D7) but expressed higher levels of *ctnnb1*, *ctnnb2*, *tcf7l2*, *fgf3* and *fgf10* (Fig. 6D1–D5) compared to controls (Fig. 6A1–A7), suggesting that *fgfr1* might restrict Wnt signaling in the developing neuromast.

**Fig. 7:**
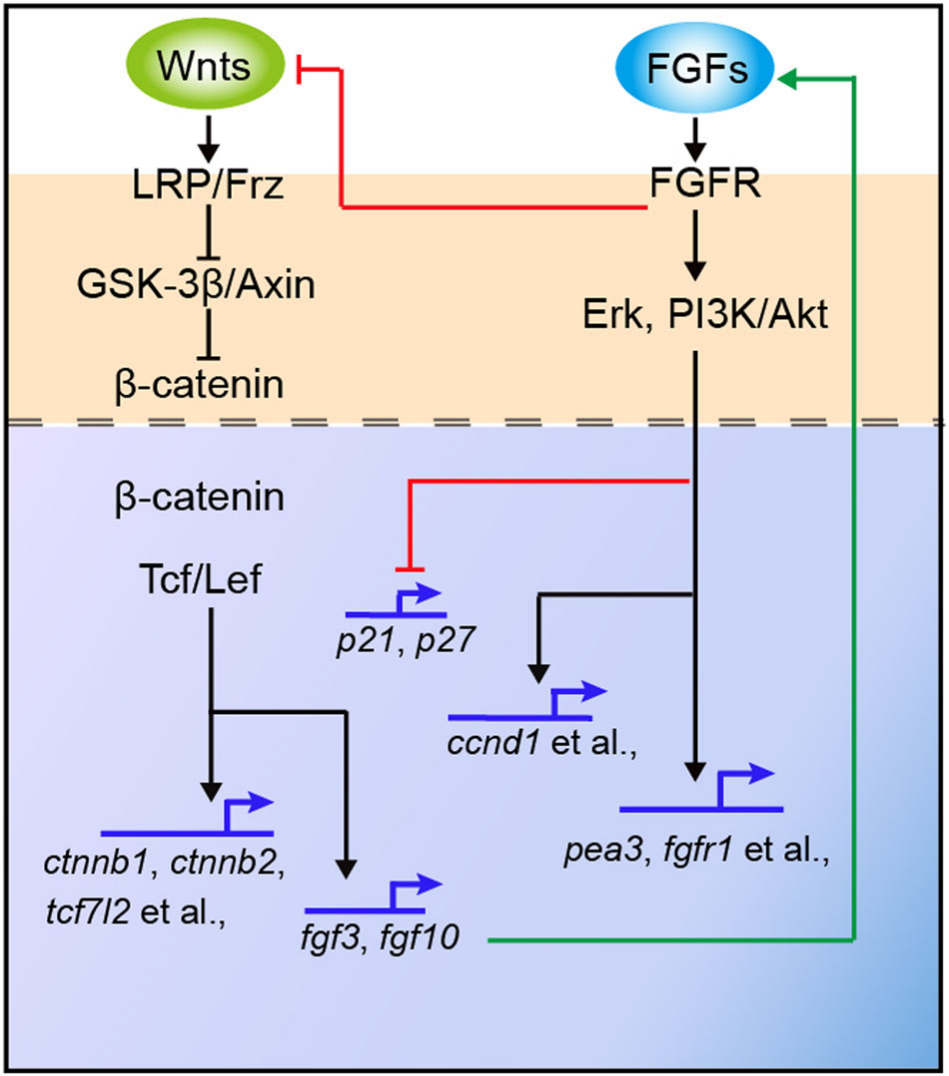
A simple model of how the Wnt and FGF signaling pathways control cell proliferation in the zebrafish neuromast. Red lines show inhibition, and green lines indicate activation

To more clearly understand the relationship between Wnt and FGF, we analyzed the expression of Wnt and FGF target genes in the experimental sets with BIO, BIO + SU5402, IWR-1, and IWR-1 + bFGF. Expression of the FGF target genes *pea3* and *fgfr1* was significantly lower in BIO+SU5402 larvae than in larvae treated with BIO alone (Fig. 6F6–F7 and B6–B7, respectively), suggesting that FGF signaling is disrupted in the presence of the FGF inhibitor; however, the levels of *ctnnb1*, *ctnnb2*, and *tcf7l2* in the BIO+SU5402-treated neuromasts were slightly higher than the levels in the BIO-treated larvae without FGF inhibition (Fig. 6F1–F3 and B1–B3). In the IWR-1 + bFGF group, there was increased expression of the FGF target genes (*pea3* and *fgfr1*) (Fig. 6G6–G7 and C6–C7), whereas no changes were detected for Wnt target gene expression across the sensory domain compared with the expression in the IWR-1–alone group (Fig. 6G1–G3 and C1–C3). These results further confirmed that SU5402-mediated *fgfr1* inhibition might induce Wnt activation. Interestingly, the expression of *fgf3* and *fgf10* in BIO+SU5402 larvae was greatly increased, which could be caused by the presence of BIO (Fig. 6F4–F5 and B4–B5). In parallel, inhibition of Wnt signaling by IWR-1 treatment during neuromast development greatly reduced *fgf3* and *fgf10* expression (Fig. 6C4–C5), but bFGF treatment could not rescue their reduction in the neuromast (Fig. 6G4–G5). Taken together, these results indicate that *fgf3* and *fgf10* expression is dependent on Wnt activity during neuromast development (Fig. 7).

### Wnt and FGF signaling are integrated in HC regeneration after neomycin damage

**Fig. 8:**
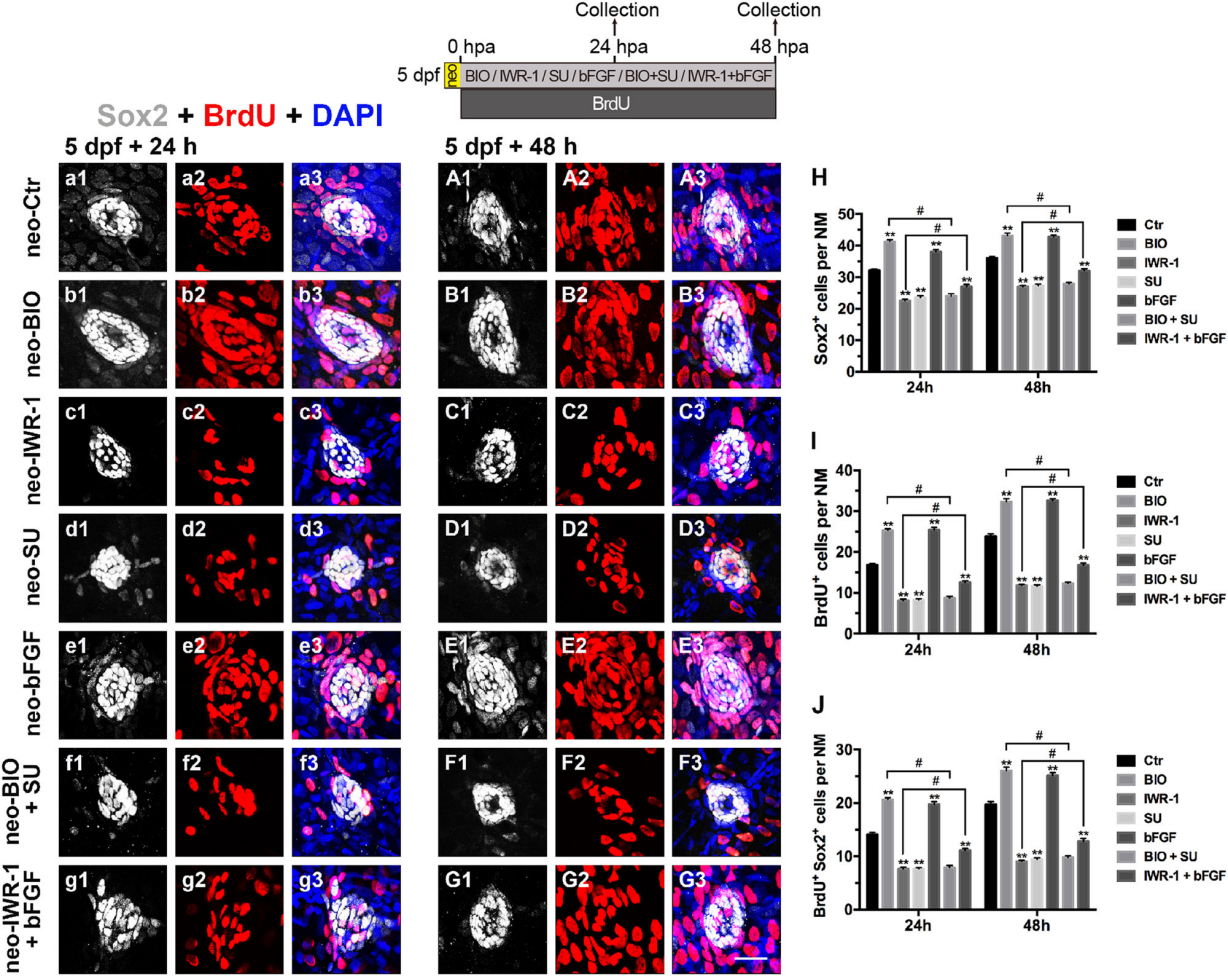
Effects of exogenous regulation of the Wnt and FGF pathways on regenerative proliferation at 24 hpa and 48 hpa after neomycin injury. **a1**–**G3**: Regenerating cells labeled with BrdU (red) and SCs labeled with Sox2 (white). **a1**–**a3**: 24 hpa after neomycin damage; **A1**–**A3**: 48 hpa after neomycin damage; **b1**–**b3**: 24 hpa after addition of BIO, an activator of the Wnt pathway; **B1**–**B3**: 48 hpa after addition of BIO; **c1**–**c3**: 24 hpa after addition of IWR-1, an inhibitor of the Wnt pathway; **C**1–**C3**: 48 hpa after addition of IWR-1; **d1**–**d3**: 24 hpa after addition of SU5402, an inhibitor of the FGF pathway; **D1**–**D3**: 48 hpa after addition of SU5402; **e1**–**e3**: 24 hpa after addition of bFGF, an activator of the FGF pathway; **E1**–**E3**: 48 hpa after addition of bFGF; **f1**–**f3**: 24 hpa after addition of BIO and SU5402; **F1**–**F3**: 48 hpa after addition of BIO and SU5402; **g1**–**g3**: 24 hpa after addition of IWR-1 and bFGF; **G1**–**G3**: 48 hpa after addition of IWR-1 and bFGF. **h**–**j**: Quantification of the supporting cells (Sox2^+^) (**h**), proliferative cells (BrdU^+^) (**i**) and regenerating supporting cells (BrdU^+^ Sox2^+^) (**j**) after blocking or enhancing the Wnt and FGF pathways. *n* = 7 fish per group. ** Indicates *p* < 0.001, ^#^ indicates *p* < 0.05, and error bars indicate the standard error of the mean. Scale bar in **G3** = 20 μm for **a1**–**G3**

**Fig. 9:**
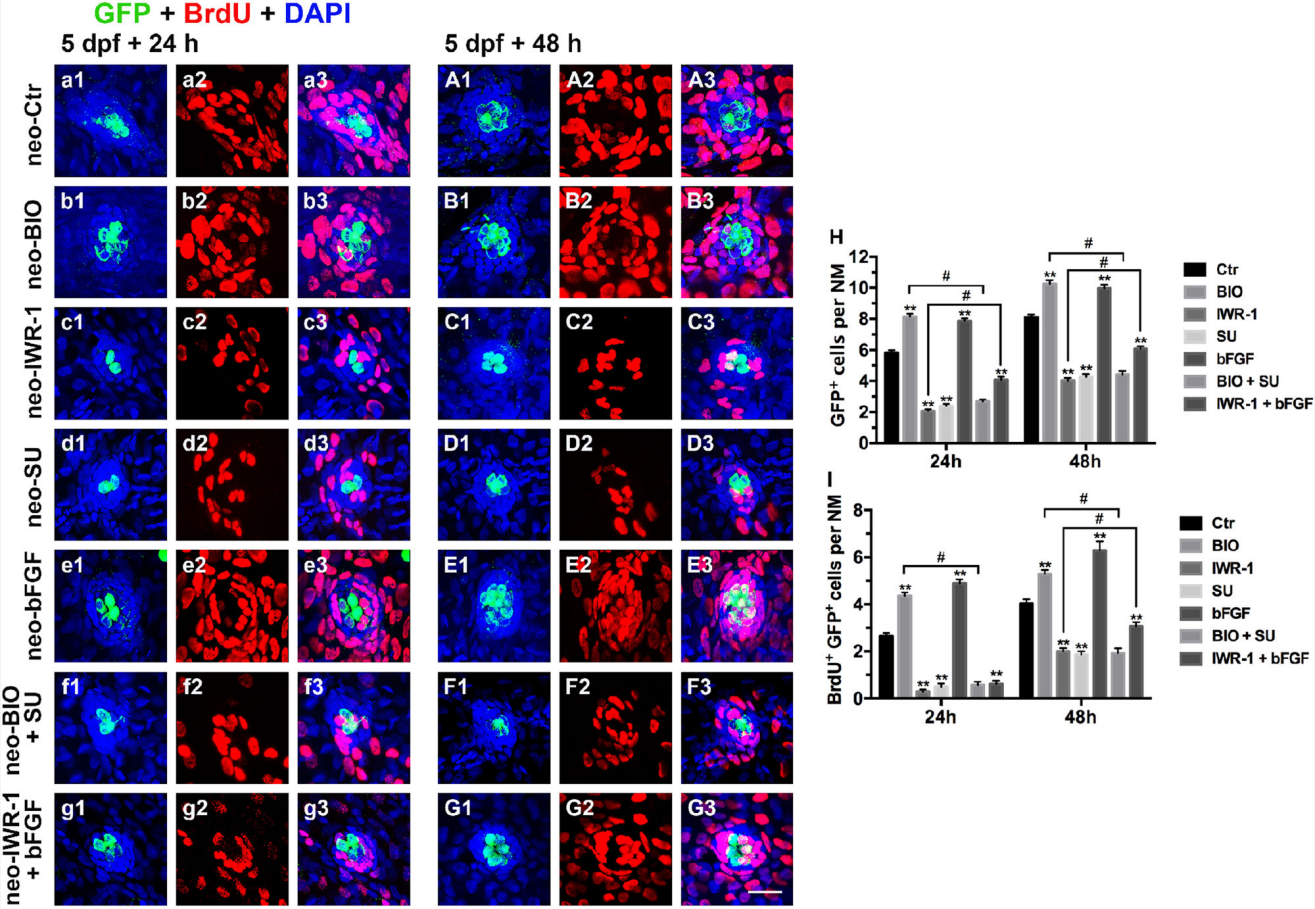
Effects of exogenous regulation of the Wnt and FGF pathways on HC regeneration at 24 hpa and 48 hpa after neomycin injury. **a1**–**G3**: Regenerating cells labeled with BrdU (red) and HCs labeled with GFP (green). **a1**–**a3**: 24 hpa after neomycin damage; **A1**–**A3**: 48 hpa after neomycin damage; **b1**–**b3**: 24 hpa after addition of BIO; **B1**–**B3**: 48 hpa after addition of BIO; **c1**–**c3**: 24 hpa after addition of IWR-1; **C1–C3**: 48 hpa after addition of IWR-1; **d1**–**d3**: 24 hpa after addition of SU5402; **D1**–**D3**: 48 hpa after addition of SU5402; **e1**–**e3**: 24 hpa after addition of bFGF; **E1**–**E3**: 48 hpa after addition of bFGF; **f1**–**f3**: 24 hpa after addition of BIO+SU5402; **F1**–**F3**: 48 hpa after addition of BIO + SU5402; **g1**–**g3**: 24 hpa after addition of IWR-1 + bFGF; **G1**–**G3**: 48 hpa after addition of IWR-1 + bFGF. **h** and **i**: Quantification of the HCs (GFP^+^) (**h**) and regenerating HCs (BrdU^+^ GFP^+^) (**i**) after blocking or activating the Wnt and FGF pathways. *n* = 7 fish per group. ** Indicates *p* < 0.001, ^**#**^ indicates *p* < 0.05, and error bars indicate the standard error of the mean. Scale bar in **G3** = 20 μm for **a1**–**G3**

Wnt and FGF signaling control progenitor cell proliferation and HC differentiation. To further confirm the roles of both signaling pathways during HC regeneration, we killed the hair cells by incubating 5 dpf *tg*(*Brn3c:mGFP*) larvae with 400 μM neomycin for 1 h, followed by incubation in 6-well plates with or without the presence of different agents for a period of 24 h or 48 h (24 hpa and 48 hpa). We then performed a BrdU (10 mM) incorporation assay and quantified the number of regenerated HCs. The BIO-treated larvae showed significantly more BrdU incorporation in the neuromasts than did DMSO-treated controls at both 24 and 48 hpa (Fig. 8a2–a3, A2–A3, b2–b3, B2–B3, i; Fig. 9a2–a3, A2–A3, b2–b3, B2–B3). Accordingly, the BIO-treated larvae had more HCs and SCs per neuromast at both 24 hpa and 48 hpa (Fig. 8a1, A1, b1, B1, h; Fig 9a1, A1, b1, B1, h). After BIO treatment, the numbers of Sox2^+^/BrdU^+^ and GFP^+^/BrdU^+^ cells were both significantly greater than in the controls (Fig. 8j and Fig. 9i). In contrast, IWR-1-treated larvae had significantly fewer proliferating cells, HCs, and SCs in the regenerating neuromast than did controls (Fig. 8c1–c3, C1–C3, h, i; Fig. 9c1–c3, C1–C3, h). The numbers of Sox2^+^/BrdU^+^ and GFP^+^/BrdU^+^ cells were also significantly lower after administration of IWR-1 (Fig. 8j and Fig. 9i). All of these results are consistent with previous findings, suggesting that Wnt signaling induces regeneration in pLL neuromasts.

Loss of FGF signaling also resulted in a significant decrease in HC regeneration, as indicated by the significant loss of BrdU^+^ regenerative cells, Sox2^+^ SCs, and GFP^+^ HCs at both 24 hpa and 48 hpa (Fig. 8d1–d3, D1–D3, h, i; Fig. 9d1–d3, D1–D3, h). To further study the role of the FGF signaling pathway during HC regeneration, we added bFGF to the fish water and found a significant increase in the numbers of proliferative cells, HCs, and SCs compared with those in DMSO-treated control neuromasts (Fig. 8e1–e3, E1–E3, h, i; Fig. 9e1–e3, E1–E3, h). The number of cells double-labeled for Sox2 and BrdU or GFP and BrdU also significantly increased after the addition of bFGF (Fig. 8j and Fig. 9i). These results indicate that FGF signaling has a significant effect on regeneration and HC production after neomycin exposure.

To determine if the increase in proliferation after Wnt activation during regeneration requires FGF signaling, we simultaneously activated Wnt signaling and inhibited FGF signaling using BIO and SU5402, respectively. Indeed, the BIO-induced increase in total proliferation was reduced to below normal levels after the simultaneous addition of SU5402, indicating that the majority of extra BrdU^+^ cells formed after BIO treatment were likely due to an increase in FGF signaling (Fig. 8f2–f3, F2–F3, i; Fig. 9f2–f3 and F2-F3). Because simultaneously activating Wnt and inhibiting FGF resulted in fewer proliferative cells compared to activating Wnt alone, we tested whether HC production was also affected by measuring the number of GFP^+^ HC cells in the larval neuromasts. We found significant differences in the numbers of GFP^+^ HCs and GFP^+^/BrdU^+^ double-positive cells between BIO+SU5402 and BIO-alone larvae (Fig. 9f1, F1, h, i). A reduction in the total number of SCs and Sox2^+^/BrdU^+^ cells in BIO+SU5402-treated larvae was also evident at 24 hpa and 48 hpa compared with that in the BIO-alone group (Fig. 8F1, F1, h, j). This conclusion is supported by bFGF-induced FGF activation in Wnt-deficient larvae. Ablation of Wnt signaling with IWR-1 caused a reduction in proliferation that could be partly rescued by simultaneous activation of FGF with bFGF at 48 hpa (Fig. 8g2, G2, i; Fig. 9g2, G2), but there were still fewer BrdU^+^ cells per neuromast than in the neomycin-alone controls (Fig. 8i; **p < 0.001). Loss of HCs and SCs following inhibition of Wnt signaling was also partly restored by ectopic FGF activation (Fig. 8g1–g3, G1–G3, h; Fig. 9g1–g3, G1–G3, h).

## Discussion

In the present study, we used a zebrafish model to gain new insights into the mechanism underlying the control of cell proliferation and fate determination in developing and regenerating neuromasts in the zebrafish pLL, and we provide evidence that activation of the Wnt and FGF pathways is crucial for promoting the proliferation and regeneration of HCs in the pLL. Our current study represents the first analysis indicating that Wnt acts through the FGF signaling pathway to promote progenitor cell proliferation and regenerative cell proliferation in the zebrafish pLL neuromasts. These findings provide strong evidence that manipulation of the Wnt and FGF pathways will be useful for promoting HC regeneration in the mammalian inner ear.

Recent studies in zebrafish showed that activating Wnt signaling promotes the proliferation of neuromast progenitors and HC regeneration in both developing and damaged neuromasts;^33,34^ however, the exact mechanisms through which Wnt signaling stimulates cell proliferation and regeneration under these conditions remain unresolved. Here, we used BIO as a Wnt agonist to activate Wnt signaling in zebrafish neuromasts. Consistent with previous reports, our results indicate that activation of Wnt/β-catenin signaling promotes cell proliferation in developing and regenerating neuromasts. More HCs and SCs were observed in the neuromasts of the BIO-treated group, which suggests that both HCs and SCs were being overproduced and that most of the proliferated SCs differentiated into HCs after BIO treatment. Genetic activation of Wnt/β-catenin signaling phenocopies the pharmacological activation by promoting progenitor cell proliferation and regenerative cell proliferation in neuromasts.

A number of studies have indicated the importance of FGF in inner ear development in many species^35^. Loss of FGF signaling in *fgf3* and *fgf8* double-deficient zebrafish not only causes failure of placode formation but also blocks HC development^36^, and Fgf3 and Fgf8 have been shown to act as upstream activators of the *atoh1* gene, which is necessary for HC development during distinct inner ear developmental periods, including both the early development of the preotic placode and the later development of the otic vesicle^37^. Based on these studies, we hypothesized that early neuromast development requires the FGF signaling pathway. To test this hypothesis, we first performed in situ analyses and confirmed the expression of candidate ligands and receptors of the FGF signaling pathway in the neuromast, revealing that this pathway is potentially activated in the developing neuromast. Next, to test whether FGF signaling is involved in the proliferation of neuromast progenitors, we inhibited the FGF signaling pathway using SU5402. The results of our proliferation assay suggested that blockade of FGF signaling inhibited cell proliferation, similar to that observed when the Wnt signaling pathway was inactivated. Given that many similarities between developmental and regenerative processes in the pLL have been identified^2,5,38–40^, we hypothesized that FGF activation would enhance regenerative proliferation and subsequent new HC generation in the pLL neuromast, similar to the effects of FGF activation during initial development. As hypothesized, neomycin-damaged larval zebrafish exposed to bFGF during recovery periods had significantly more SCs in their neuromasts than neomycin-alone control animals, indicating that increased cell proliferation had occurred, and this was followed by increased HC production. Together, these results suggest that FGF signaling plays a key role in regulating normal neuromast development and HC regeneration by promoting proliferation, similar to that observed for Wnt signaling in HC development and regeneration.

To identify the Wnt-FGF interactions that are required for cell proliferation in larval zebrafish neuromasts, we performed epistasis experiments using a combination of pharmacological and genetic approaches to manipulate Wnt and FGF activity. We compared cell proliferation with activated Wnt signaling alone or with activation of Wnt and inhibition of FGF signaling using the small molecule inhibitor SU5402. While activation of Wnt alone increased cell proliferation and induced greater HC numbers, it was unable to do so in the absence of FGF signaling. To further confirm that FGF functions downstream of Wnt signaling to promote cell proliferation in developing neuromasts, we used IWR-1 to downregulate the Wnt signal and then added bFGF to activate the FGF pathway. Our proliferation assay showed that bFGF treatment encouraged neuromast progenitors to proliferate and partly rescued the cell proliferation phenotype in the absence of Wnt signaling. In neomycin-damaged neuromasts, simultaneous manipulation of Wnt and FGF signaling activities showed that while Wnt signaling is sufficient to drive ectopic cell proliferation and subsequent HC regeneration, this pathway was able to do so only in the presence of functional FGF signaling.

In this work, we characterized the expression patterns of the FGF and Wnt pathway components in developing neuromasts in detail. We showed that Wnt activation significantly increased the expression of FGF components such as *fgf3*, *fgf10*, *pea3*, and *fgfr1*, whereas their expression in IWR-1-induced Wnt-inhibited neuromasts was significantly reduced. Furthermore, stimulating FGF signaling through the addition of bFGF had no effect on the expression of Wnt components. In addition, no significant changes in the expression of *ctnnb1, ctnnb2*, or *tcf7l2* were observed in the neuromasts of the IWR-1+bFGF group compared to that in the neuromasts of the IWR-1–alone group, indicating that there had been no reactivation of Wnt signaling by bFGF treatment after IWR-1 treatment. Taken together, our results suggest that the pro-proliferative effect induced by Wnt activation was mainly mediated by the FGF pathway, which is consistent with previous reports. For instance, *Fgf8* levels are reduced in the absence of Wnt activity in mouse embryos^41^, while FGF activity is enhanced in β-catenin overexpression embryos^42,43^, suggesting that FGF functions downstream of Wnt/β-catenin signaling. Intriguingly, FGF activation did not change the expression of *fgf3* or *fgf10* in developing neuromasts, and thus, Wnt can be reasonably assumed to activate the FGF signaling pathway through *fgf3* and *fgf10*. We also found that inhibiting FGF signaling with SU5402 resulted in downregulation of *fgfr1* and upregulation of *fgf10* and *fgf3* as well as several Wnt components, suggesting that a feedback mechanism involving Fgfr1 might operate during neuromast proliferation. A number of studies have shown that FGF components, such as *fgf3* and *fgf10a*, have mitogenic activity;^44–46^ however, their signaling functions in cell proliferation during neuromast development have not yet been explored. We showed that active Wnt and FGF signaling exerted their function by negatively regulating the cell cycle inhibitor genes *p21* and *p27* and positively regulating the *ccnd1* gene to permit cell proliferation. Sequential induction of Wnt signaling and inhibition of FGFR caused a complete loss of *ccnd1* expression and enhanced *p21* and *p27* expression in the neuromast, similar to SU5402 treatment alone, indicating an essential role of FGF signaling in maintaining the expression of cell cycle genes.

In this study, we showed that the Wnt and FGF pathways cooperate closely to regulate proliferation in the zebrafish neuromast. To fully understand the heterogeneity of the neuromast domain, it is necessary to identify all of the transcriptional targets that are activated or repressed by FGF and Wnt regulation during neuromast proliferation in zebrafish. Recently, many studies have shown that bFGF activates several intracellular pathways, including the RAS/ERK and PI3K/AKT cascades^47–49^. Therefore, defining which signals act downstream of the Wnt-FGF interactions during proliferation in neuromasts is of great interest.

In summary, we have investigated the cross talk between the Wnt and FGF signaling pathways during cell proliferation and regeneration in the zebrafish neuromast and have shown that FGF activation enhances proliferation and promotes subsequent cell differentiation in the developing and regenerating neuromasts of the zebrafish pLL. Furthermore, we provide evidence that Wnt signaling activates FGF activity via *fgf3* and *fgf10* stimulation and that FGF signaling inhibits the Wnt pathway through the Wnt inhibitor *fgfr1*. Our findings contribute to studies on HC biology and provide a new avenue for understanding the signaling mechanisms that regulate cell proliferation during mammalian inner ear development.

## Supplementary information

Supplementary Figure Legend

Supplementary Figure 1

Supplementary Figure 2

Supplementary Figure 3

Supplementary Table S1
