# Supplementary material for: Wnt/β-catenin interacts with the FGF pathway to promote proliferation and regenerative cell proliferation in the zebrafish lateral line neuromast
Source: Exp Mol Med. 2019 May 23;51(5):1–16. doi: 10.1038/s12276-019-0247-x (PMC6533250; doi:10.1038/s12276-019-0247-x)
Supplement: Supplementary file 1 — Supplementary Figure Legend [file 12276_2019_247_MOESM1_ESM.docx]

**Wnt/β-catenin interacts with the FGF pathway to promote proliferation and regenerative cell proliferation in the zebrafish lateral line neuromast**

**Supplementary figure legends**

**Supplementary Fig 1. Effects of exogenous regulation of Wnt on the expression of Wnt pathway genes.** A1–C3: WISH analysis of *ctnnb1* (A1–C1), *ctnnb2* (A2–C2), and *tcf7l2* (A3–C3) expression in the neuromasts from different groups. Scale bar in C3 = 30 μm for A1–C3.

**Supplementary Fig 2. Effects of exogenous regulation of FGF on the expression of FGF pathway genes.** A1–C2: WISH analysis of *pea3* (A1–C1) and *fgfr1* (A2–C2) expression in the neuromasts from different groups. Scale bar in C2 = 30 μm for A1–C2.

**Supplementary Fig 3. The effects of** **BIO, IWR-1, SU5402, bFGF, BIO + SU5402, and IWR-1 + bFGF on apoptosis in zebrafish larvae at 72 hpf.** Apoptosis was analyzed with a TUNEL assay (green).
