# Supplementary figures and images for: Wnt/β-catenin interacts with the FGF pathway to promote proliferation and regenerative cell proliferation in the zebrafish lateral line neuromast
Source: Exp Mol Med. 2019 May 23;51(5):1–16. doi: 10.1038/s12276-019-0247-x (PMC6533250; doi:10.1038/s12276-019-0247-x)

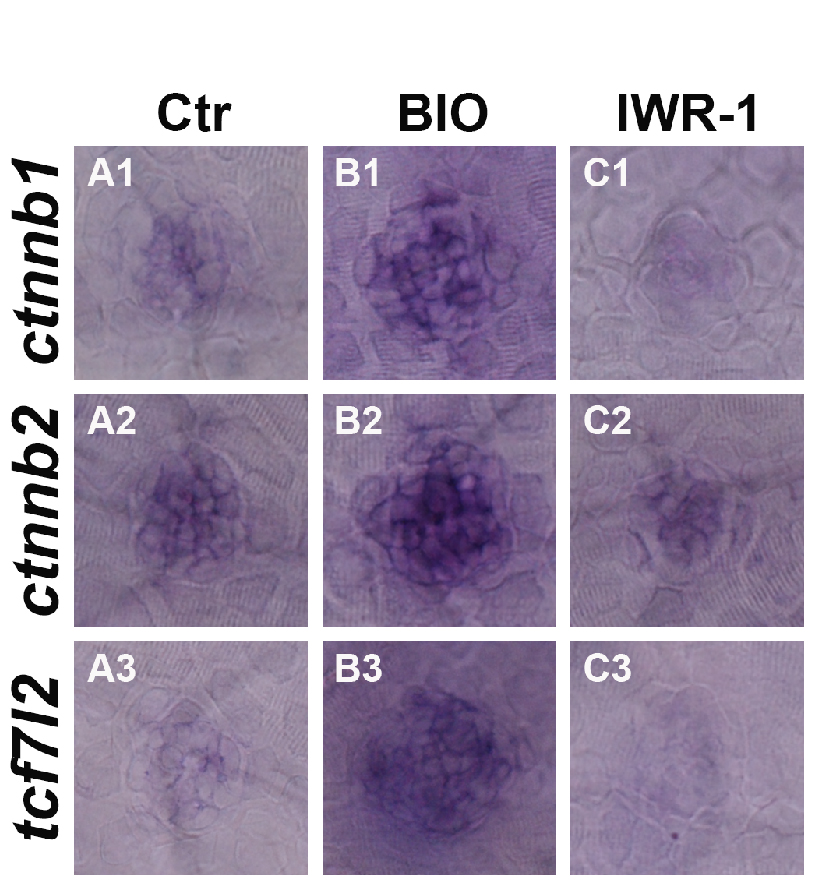

Supplement: Supplementary file 2 — Supplementary Figure 1 [file 12276_2019_247_MOESM2_ESM.jpg]

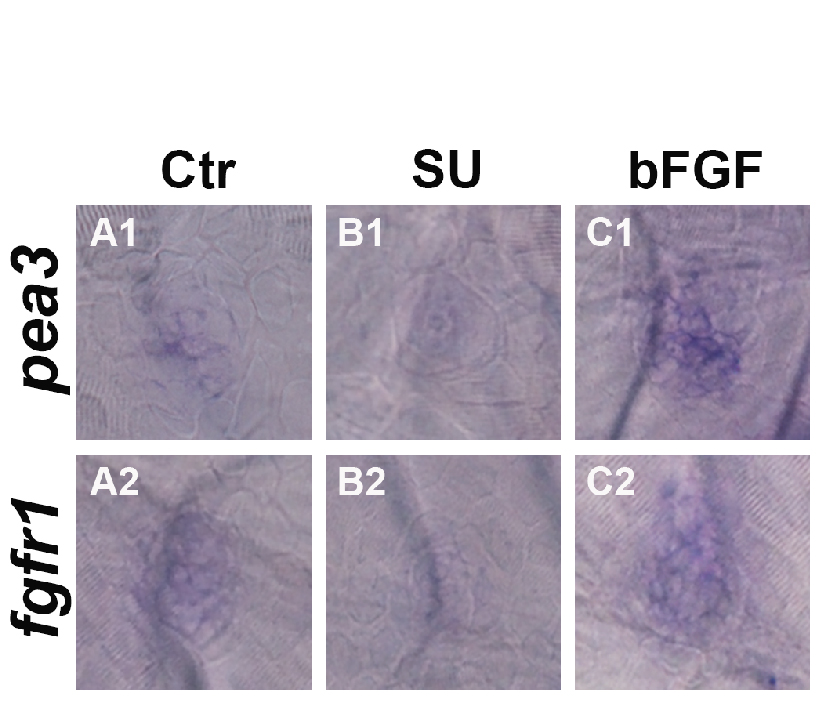

Supplement: Supplementary file 3 — Supplementary Figure 2 [file 12276_2019_247_MOESM3_ESM.jpg]

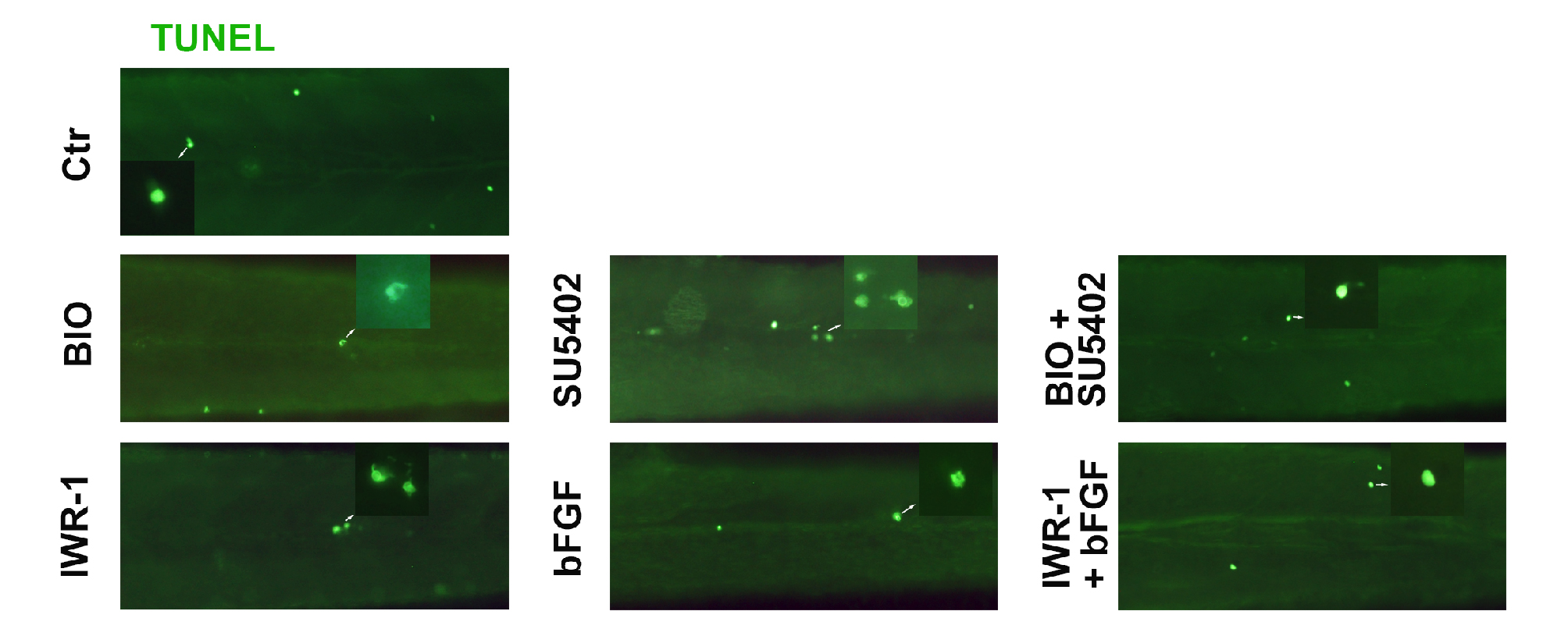

Supplement: Supplementary file 4 — Supplementary Figure 3 [file 12276_2019_247_MOESM4_ESM.jpg]
