# Supplementary material for: Wnt/β-catenin interacts with the FGF pathway to promote proliferation and regenerative cell proliferation in the zebrafish lateral line neuromast
Source: Exp Mol Med. 2019 May 23;51(5):1–16. doi: 10.1038/s12276-019-0247-x (PMC6533250; doi:10.1038/s12276-019-0247-x)
Supplement: Supplementary file 5 — Supplementary Table S1 [file 12276_2019_247_MOESM5_ESM.docx]

**Table S1. Primers used in the study.**

| **Gene Name** | **Forward Primer** | **Reverse Primer** |
| --- | --- | --- |
| ***ctnnb1*** | 5ʹ-cccaggactacaagaagcga-3ʹ | 5ʹ-acaggcaaggctaaggttga-3ʹ |
| ***ctnnb2*** | 5ʹ-catcgagaacatccagcgtg-3ʹ | 5ʹ-tggactacactacagccgtc-3ʹ |
| ***tcf7l2*** | 5ʹ-ccctccacatctacagggag-3ʹ | 5ʹ-tgtgttcattgccctctcct-3ʹ |
| ***fgf3*** | 5′-tgagcttcttggatccgagt-3′ | 5′-tgccgctgactctctctaag-3′ |
| ***fgf10*** | 5′-ctgctgcttctgttcctgtg-3′ | 5′-agtgccttcttctccaaatgg-3′ |
| ***pea3*** | 5′-agtgtgtttcgtgaaggtgc-3′ | 5′-atacaagaggatggggtggg-3′ |
| ***fgfr1*** | 5′-gtatctcgcatccaagaagtgt-3′ | 5′-agctgtatgtgtttctcccaga-3′ |
| ***atoh1a*** | 5′-gtcaaagtacgcgagctctg-3′ | 5′-acttcagtgaggcgagaact-3′ |
| ***Sox2*** | 5′-cgcatatgaacggctggac-3′ | 5′-tgtgttctttccttgagctct-3′ |
| ***p27kip1*** | 5′-acttcgacttttccacgcac-3′ | 5′-tgctttattgttgagtgccaga-3′ |
| ***p21*** | 5′-acaagcggatcctacgttca-3′ | 5′-ctacgagacgaatgcagctc-3′ |
| ***ccnd1*** | 5′-tgctcgaggtctgtgaagag-3′ | 5′-tctgtgggagtgcaagagag-3′ |
